# Treating Periodontitis Strictly Non-Surgically—A Retrospective Long-Term Analysis of Tooth Loss During Supportive Periodontal Care

**DOI:** 10.3390/dj13040146

**Published:** 2025-03-27

**Authors:** Marco Michael Herz, Sandra Braun, Nina Hoffmann, Stefan Lachmann, Valentin Bartha, Hari Petsos

**Affiliations:** 1 Department for Conservative Dentistry and Periodontology, Tuebingen University, 72076 Tuebingen, Germany; 2 Private Practice, 72336 Balingen, Germany; s.braun@student.uni-tuebingen.de; 3 Private Practice, 76189 Karlsruhe, Germany; nh@za-hoffmann.de; 4 Private Practice, 72072 Tuebingen, Germany; stlachmann@t-online.de; 5 Department for Conservative Dentistry, Heidelberg University, 69120 Heidelberg, Germany; valentin.bartha@med.uni-heidelberg.de; 6 Department of Periodontology, Center of Dentistry and Oral Medicine (Carolinum), Goethe University Frankfurt, 60596 Frankfurt am Main, Germany; petsos@med.uni-frankfurt.de; 7 Private Practice, 59494 Soest, Germany

**Keywords:** periodontal tooth loss, periodontitis therapy, periodontal risk factors, supportive periodontal care

## Abstract

**Background/Objectives:** Retrospective analysis of long-term periodontal tooth loss (PTL) during supportive periodontal care (SPC) in patients with Stage III/IV periodontitis who received strictly non-surgical periodontal treatment. **Methods:** Fully documented medical documentation of SPC > 5 years was analyzed at T0 (baseline), T1 (after Steps 1/2), and during SPC (T2). PTL, periodontal pocket depth (PD), bleeding on probing (BOP), tooth mobility (TM), furcation involvement (FI), and frequency of SPC were recorded. Each parameter was tested for significance in a bivariate analysis, before a multilevel logistic regression analysis was performed to identify possible factors with an impact on PTL during SPC. **Results:** A total of 51 women/64 men (T1 mean age 55.4 ± 10.3 yrs) were surveilled after 9.0 ± 2.4 yrs; 2647 teeth were included. On average, patients attended 10.6 ± 3.8 SPC sessions between T1 and T2; 77 patients (67%) attended at least 1/year. At T1, 68 teeth were lost; 6.1% of the remaining teeth showed FI, and 13.8% showed TM. During SPC, the PTL range was 118 (1.03 ± 1.21/patient). TM, FI, mean PD, and Stage IV periodontitis proved to be statistically significantly associated with increased PTL. **Conclusions:** PTL was low in this cohort. Nevertheless, at T1, it may be beneficial to focus on stopping TM by splinting the mobile teeth and reducing the PD or treating FI appropriately, understanding that these precise applications of surgical procedures could positively affect long-term tooth retention.

## 1. Introduction

Periodontal therapy focuses not only on limiting inflammatory processes, but also on ensuring minimal periodontal tooth loss (PTL) during the treatment period [1,2,3]. The loss of one or more teeth can have potentially serious consequences for the patient, including significant detriments to esthetics, chewing, and phonetics [4,5]. Measures to address these issues are almost always associated with high costs for the patient, the health insurance company, or both [5,6,7,8,9].

Long-term supportive periodontal care (SPC) is meant to maintain the periodontal condition achieved during Steps 1 to 3 of periodontal therapy [3]. During SPC visits, the patient’s compliance is reviewed using periodontal indices, the patient continues to be motivated and re-instructed, a detailed periodontal status is recorded, and supragingival and subgingival tooth cleaning is performed [3].

Some important periodontal risk factors for tooth loss have been known for a long time. Smoking, for example, can increase the risk of tooth loss by a factor of 3 [10], while insufficiently controlled diabetes mellitus type 2 increases the risk by a factor of 1.2–1.9 [11].

Several studies have addressed the problem of PTL, especially during SPC [12,13,14,15]. These studies variously associate both patient- and tooth-related factors with PTL. At the patient level, older age at the onset of SPC, smoking, compliance with SPC, an initial diagnosis of periodontitis, systemic diseases, insurance status, and marital and educational status are factors influencing PTL. Factors at the tooth level include tooth mobility (TM), furcation involvement (FI), molars in general, clinical attachment loss (CAL), bleeding on probing (BOP), the mean periodontal pocket depth (PD), and residual periodontal pockets ≥ 6 mm [13,16,17,18]. These observational studies generally report the use of surgical periodontal therapy by specialized periodontists after Step 2 of periodontal therapy when residual deep pockets were still present.

C.H. Drisko stated in her 2001 review that “non-surgical therapy remains the cornerstone of periodontal treatment” [19]. According to M. Aimetti, non-surgical periodontitis treatment primarily serves to control microbial infection in the pocket, which is achieved by clearing the root surfaces of bacterial biofilm, calculus, and bacterial toxins [20]. In Germany, dentists perform non-surgical periodontal therapy in a vast number of cases. This is in line with the minimum therapy required based on the European Federation of Periodontology (EFP) guidelines [3] and the regulations of statutory health insurance funds. This fact is also clearly supported by the strikingly disproportionate ratio of non-surgical to surgical billing items across insurance providers, regardless of the patient’s insurance status [21].

Studies investigating factors associated with PTL in the setting of non-surgical periodontal therapy are lacking. Therefore, this retrospective cohort study aimed to investigate long-term PTL during SPC after strictly non-surgical periodontal treatment, and to identify factors potentially associated with PTL.

## 2. Materials and Methods

### 2.1. Study Design and Inclusion Criteria

The present retrospective cohort study was based on the analysis of data from patients treated for periodontitis between 1994 and 2018.

**Figure 1:**
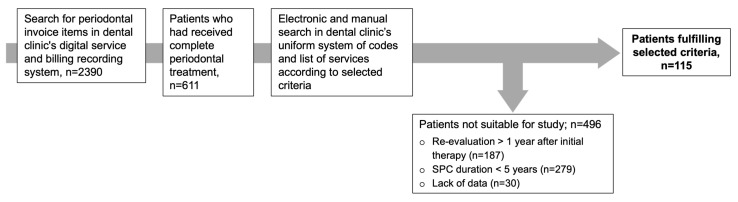
Patient selection.

This observational study considered the STROBE (Strengthening the Reporting of Observational Studies in Epidemiology) recommendations, and ensured compliance with the standards for reporting observational studies set out therein. The patients were identified through electronic database searches at the Department for Conservative Dentistry at the University Hospital of Tuebingen, and selected according to the following criteria (Figure 1):

- Completed active periodontal therapy without periodontal surgery;
- Periodontitis Stage III/IV;
- Complete examination data for baseline (T0), re-evaluation (T1) ≤ 1 year after T0, and SPC (T2 = last SPC visit);
- SPC duration ≥ 5 years;
- No adjuvant antibiotic treatment at any time;
- Reason for tooth loss clearly documented.

### 2.2. Primary and Secondary Outcomes

The primary outcome of this study was the tooth loss (TL) rate during SPC. Tooth- and patient-level factors that might be associated with tooth loss during SPC constituted the secondary outcomes.

The reasons for tooth loss (periodontal or non-periodontal) were documented. Non-periodontal reasons included non-restorable carious lesions, trauma, endodontic complications, and root fractures (longitudinal and transverse). PTL, however, was defined as TL caused by a combination of progressive CAL, FI II/III, and/or TM II/III.

### 2.3. Clinical Procedures and Time Intervals

**Figure 2:**
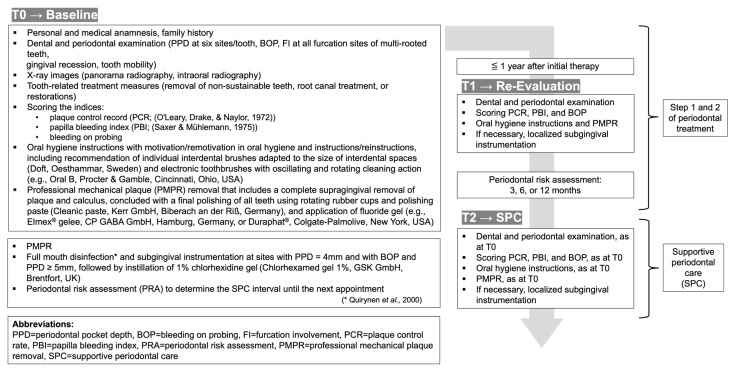
Clinical procedures for periodontal treatment at T0–T2 [22,23,24].

All patients underwent a standardized treatment protocol (Figure 2). The protocols of Step 2 (re-evaluation) and Step 4 (SPC) were identical to the treatment protocol of Step 1 (baseline). This means that detailed data must be collected at every appointment regarding dental and periodontal examination, plaque control record (PCR) scoring, the papilla bleeding index (PBI), BOP, oral hygiene instructions, professional mechanical plaque removal (PMPR), and, if necessary, subgingival debridement.

### 2.4. SPC Attendance

As different recall times were noted for some patients, categorization into regular and irregular appointments had to be performed as follows:

- SPC ≥ 1 visit/year = regular recall/SPC
- SPC < 1 actual visit/year = irregular recall/SPC

### 2.5. Data Recorded for the Study

The following data were extracted from the medical records:

- Patient characteristics: age, gender, smoking behavior (active smoker, former smoker [non-smoker for 5 years or more], non-smoker), and diabetes mellitus without HbA1c (glycated hemoglobin) value (because this value was inconsistently documented);
- Periodontal parameters: PD, BOP, FI, TM, CAL, and initial periodontitis stage/grade;
- Tooth characteristics: tooth type (single-/multi-rooted) and number of lost teeth;
- Therapy characteristics: SPC (number of visits and duration), abutment status (fixed/removable), and duration of periodontitis therapy Steps 1 and 2

All patients received strictly non-surgical treatment during Steps 1 and 2 of periodontal therapy and SPC sessions in a university setting. Steps 1, 2, and 4 (SPC) of periodontal therapy were performed either by undergraduates with varying degrees of experience under the supervision of periodontally skilled dentists, or by dental professionals. Different types of dentures were categorized, regardless of the material: fixed restorations, including crowns and bridges; removable dentures, including clasp dentures; and combined dental work, such as using telescopic dentures. The present cohort has already been investigated in relation to another research question [25]. Hence, the applied clinical methodology and parts of the research methodology have been described previously.

### 2.6. Statistical Analysis

Data on all study participants at the time points T0, T1, and T2 were recorded in a pseudonymized manner in an Excel-based data matrix (Microsoft Excel for Windows, Microsoft Corporation, Redmond, WA, USA). The primary outcome parameter was defined as changes related to TL/PTL during SPC, where patients were considered statistical units. Data at the patient level were described using absolute (mean ± standard deviation (SD)) and/or relative (%) frequencies, while data at the tooth level were described separately for T0, T1, and T2 using absolute and relative frequencies. Univariate correlations of variables at the patient level were calculated using the chi-square test or Pearson’s correlation coefficient. Degrees of freedom and |r|-values were additionally reported in the case of significant differences. Calculations using multilevel logistic regression models, with the patient as the first level and the tooth as the second level at T1, and the overall TL/PTL as dependent variables, were used to analyze factors at the patient and/or tooth level that may influence TL/PTL. Variables with significant bivariate correlations with TL/PTL during SPC were thus included in the model.

The following variables were tested:

- At the tooth level:
  ○ At T1 (re-evaluation) and T2 (last SPC): number of teeth;
  ○ At T1: mean PD, mean CAL, abutment status, FI, TM, and surgical treatment need (at least 1 site ≥ 6 mm).
- At the patient level:
  ○ At T0 (baseline) and T1: mean BOP;
  ○ At T1: gender, age, smoking status, diabetic status, annual SPC, stage, grade, and the number of SPC appointments;
  ○ At T2: maximum PTL.

As an indicator of how well the model fitted the data, “−2 log likelihood” (−2 LL) was calculated. Third molars were excluded from the data analysis. The significance level was defined as 0.05. Statistical analyses were performed using appropriate software (IBM SPSS Statistics 29 software package, IBM, Boeblingen, Germany).

## 3. Results

### 3.1. Baseline Characteristics

Of a total of 115 patients (51 female/44%, 64 male/56%), 77 patients (67%) attended SPC visits at least once per year. A total of 53 patients (46%) lost at least one tooth over the SPC period. The mean age at the start of the SPC period was 55.4 ± 10.3 years. Twenty-five patients (22%) were smokers at T1, and diabetes mellitus was a risk factor in 12 patients (10%). Of the 115 patients, 28 were diagnosed with Stage IV periodontitis (24%) and 46 with Grade C periodontitis (40%). The SPC duration analyzed was 9 ± 2.4 years, with a total of 10.6 ± 3.8 SPC sessions per patient. At T0, the maximum periodontal bone loss was significantly greater in patients with TL than in those without TL (Table 1).

The number of teeth decreased over the observation period, with a loss of 68 teeth during Steps 1 and 2, and another 133 teeth during SPC. Regarding the SPC phase and focusing on periodontal reasons, this equates to an annual PTL rate of 0.13 ± 0.26 teeth/patient. Between T0 and T1, a total of 68 teeth were lost: 63 for periodontal reasons, 3 due to caries, and 2 due to root fractures. Regarding the 133 teeth lost during SPC, 118 were lost for periodontal reasons, 3 due to endo-perio problems, 2 due to excessive caries, and the remaining 10 due to root fractures. The number of anterior teeth, premolars, and molars decreased statistically significantly up to T2. The same applies to the categorization of single-rooted versus multi-rooted teeth, as presented in Table 2.

### 3.2. Tooth Loss

PTL occurred more often in multi-rooted teeth with FI than in those without FI. A total of 392 teeth had a prosthetic restoration, of which 34 were lost. Table 3 provides an overview of the root configuration of the teeth, abutment status, and TM for both the total tooth loss and the PTL.

The parameters mentioned in the statistical analysis that are not listed in the tables did not show statistical significance in the bivariate analysis, and were therefore not included in the multilevel regression analysis. FI degrees II/III at re-evaluation revealed a significant positive association with PTL, as displayed in Table 4. TM of any degree and the mean PD at re-evaluation also showed significant associations with PTL. At the patient level, Stage IV periodontitis was significantly associated with PTL. The abutment status had no effect.

## 4. Discussion

### 4.1. General Discussion

In the present study, 115 patients, with a total of 2647 teeth, were analyzed retrospectively over an average observation period of 9.0 ± 2.4 years. During SPC, a total of 118 teeth (1.03 ± 1.21 teeth per patient) were lost for periodontal reasons, which corresponds to an annual TL of 0.13 ± 0.26 teeth per patient. This TL rate is roughly on par with the rates in several studies that include surgical treatment, if indicated. Pretzl et al. demonstrated an average TL of 2.87 teeth per patient during SPC, resulting in a mean annual TL rate of 0.14 teeth/patient over 20 years. Graetz et al. also assessed the risk of TL after Steps 1–3, which included non-surgical and, if indicated, surgical treatment in 315 patients. In their study, 351 teeth were lost during Steps 1–3, and a further 816 during SPC, with 0.15 ± 0.17 teeth being lost per patient and year overall [26]. Bäumer et al. also determined that aggressive periodontitis (the term used at the time) caused the loss of 0.63 teeth per patient during Steps 1–3, and 1.34 teeth during SPC [27]. The PTL rate of this study is therefore roughly on par with the rates in previous studies.

At re-evaluation, the multilevel analysis for PTL revealed a significant positive association with increased TM, FI, and mean PD at the tooth level and Stage IV periodontitis at the patient level.

Hirschfeld and Wassermann classified the prognosis of a periodontally compromised tooth as unfavorable in the case of increased TM and FI or deep pockets and pronounced bone loss [28]. All degrees of TM in the present study were associated with TL. The odds ratio (OR), and thus the probability of losing one or more teeth, increased with higher degrees of TM. Other studies that comprehensively included non-surgical and surgical periodontal therapy measures also reported a higher risk of PTL with increasing mobility, and identified TM as a significant and essential reason for tooth loss in general [13,16,29,30,31]. Progressive bone loss and the increasingly questionable prognosis [32,33,34] could lead to an increased rate of extractions. Supporting this, Agudio et al. noted that TM is commonly caused by pronounced bone destruction [30]. This implies that accurate diagnostics and precise classification, along with the practitioner’s level of experience, are crucial for correctly identifying any existing TM. Aminoshariae et al. have already pointed out the problem of inconsistent and imprecise classifications of the degrees of mobility, and advocate for the use of only one classification system [35].

Since TM was associated with PTL at T1 and T2, the question of whether the affected teeth should be splinted directly arises. In addition, the new EFP S3 level clinical practice guideline also includes this therapeutic measure in the treatment of Stage IV periodontitis, recommending the splinting of mobile teeth [36]. This may not only lead to higher survival rates and improved periodontal stability [37,38], but also improve chewing ability and overall oral comfort [39,40]. However, current evidence also suggests that splinting has a limited impact on traditional periodontal outcomes. A recent systematic review by Dommisch et al. (2022) evaluated the long-term efficacy of tooth splinting in periodontitis patients with masticatory dysfunction. Their primary outcome was tooth loss, with secondary outcomes including PD, CAL, and TM. Across the available studies, the review found that splinted teeth did not have significantly different long-term tooth loss rates compared to non-splinted teeth in the same patients [41]. This suggests that, from a purely clinical periodontal standpoint, splinting can be considered neutral, meaning that it does not cure disease or improve attachment outcomes per se. Hence, the benefits mentioned previously could be considered as primarily palliative, but even if this is true, they are nonetheless important in the overall management of advanced cases, as they help patients to tolerate and undergo the needed periodontal treatments, particularly in cases of teeth with high mobility.

A restoration with fixed dentures also has a certain splinting function. In comparison to fixed dentures, in this study, no statistical associations were found for teeth with prosthetic restorations, for fixed or removable dentures. It is also worth mentioning that none of the patients in this study had undergone a splinting intervention or had splinted teeth. The relationship between removable dentures and the progression of periodontitis has already been discussed in numerous studies [42,43,44]. Pretzl et al. showed that a removable construction in a periodontally compromised dentition correlated with higher PTL than a fixed restoration [32]. Preshaw et al. found no clear relationship between removable dentures and periodontitis progression [42]. Rissin et al. also found no difference between the progression of periodontitis and the type of restoration (fixed or removable) [45].

In this study, a total of 70 single-rooted teeth and 48 multi-rooted teeth, 14 of them with FI, were lost during SPC (T1–T2). The PTL for these teeth was significant for FI Degrees III and II. In several studies, multi-rooted teeth were more likely to be lost per se [17,33,46,47]. Other studies have identified existing FI as a risk factor for TL, regardless of the periodontal treatment applied [29,32,48]. The results of the present study also show that the risk of TL increases with the degree of FI, aligning with previously published results [48,49,50,51]. Dannewitz et al. also reported the highest TL rates for teeth with FI Degree III in their studies, although periodontal therapy consisted of non-surgical and surgical treatment steps [47,48].

It is usually questioned whether it is worth preserving teeth with FI Degree III, due to their often complex root anatomy with limited anatomical accessibility to the furcation, resulting in inadequate cleaning. These considerations result in a generally higher extraction rate for such teeth [52,53]. Nibali et al. stated that the treatment of teeth with FI requires precise diagnosis and classification of the clinical condition, gained through sufficient clinical experience [54]. It is interesting to note that the identified risk factors at re-evaluation for TL during SPC were consistent. This shows that the risk of losing a tooth due to TM/FI/PD or Stage IV periodontitis could not be compensated by non-surgical therapy alone in the present cohort once it was initially diagnosed.

In the present study, the mean PD was statistically significant for PTL, while a PD ≥ 6 mm was not significant as a target value, both for a possible surgical approach according to the EFP S3 guideline and for successful periodontal treatment. [3,36,55]. The proportion of periodontal pockets with a PD ≥ 6 mm at T1 was only 11.48% compared to the total number of all pockets, and was even lower in patients with PTL. Therefore, the amount of PD ≥ 6 mm may have been too small for a statistically significant result. Moreover, while our analysis was conducted at the tooth level, the categorization of PPD ≥ 6 mm as a binary variable may have limited its discriminative power, differing from studies that particularly analyzed individual teeth with deep pockets over the study period. Additionally, successful treatment of deep pockets during SPC may have modified their impact on long-term tooth retention.

For several authors, the presence of deep PD after treatment is a significant predictor of further attachment loss, which may ultimately lead to tooth loss. Werner et al. found that a deeper PD benefited from non-surgical treatment, but a PD ≥ 6 mm (11% at T1) remained deep even after treatment [56]. Herz et al. concluded that residual pockets after Steps 1 and 2 of periodontal treatment lead to the worsening of periodontal pockets [25]. Saleh et al. concluded that patients with a residual PD ≥ 5 mm at >15% (10.2%) of the sites have a higher risk of long-term tooth loss after initial treatment. However, the patients examined underwent both surgical and non-surgical treatment measures [18]. Meisel et al. also stated that the severity of PD leads to further tooth loss even after many years, depending on the depth, therefore recommending regular pocket probing to prospectively identify patients with a high risk of tooth loss in the early stages [57]. Bumm et al. performed non-surgical treatment on persistent periodontal pockets, resulting in a mean PD reduction of 1.32 ± 1.79 mm, with a medium PD = 4–5 mm responding better to treatment than a deep PD ≥ 6 mm [58]. Further recent reviews also emphasize that a PD ≥ 6 mm contributes significantly to periodontal instability and tooth loss [20,59,60].

### 4.2. Limitations of the Study

This study has some limitations that should be addressed self-critically. Some data in this study originate from a time when the etiological aspects of periodontitis that are known today and many influencing factors were not explicitly asked for, and only documented randomly, or not at all. Examples include the HbA1c value and the exact number of cigarettes per day. The inclusion of these parameters would be very useful and increase the potential significance of the study.

Moreover, the potential effects of dietary factors and lifestyle factors, such as mental stress, physical activity, and sleep quality, could not be considered, as they did not play a role in the initial records relevant to the study, and were therefore not queried. Because this study retrospectively analyzed patient chart data from two decades in a single geographic location, caution should be exercised when extrapolating the findings to other populations with different socio-economic backgrounds, healthcare systems, and periodontal treatment approaches.

The retrospective data available for analysis were partly compiled by practitioners with varying degrees of experience and without calibration. Despite adjusting the multilevel regression models, statistical interaction effects could have influenced the results to a relevant extent.

## 5. Conclusions

PTL was low in the present cohort. At the tooth level, the results of this study revealed TM, FI, and mean PD as strongly associated with PTL at re-evaluation. At the patient level, Stage IV periodontitis was associated with PTL at any time. At re-evaluation, it may be beneficial to focus on stopping TM by splinting the mobile teeth and reducing PD or treating FI appropriately, understanding that these precise applications of surgical procedures could positively affect long-term tooth retention. For this reason, the concept of strictly non-surgical periodontitis therapy must be critically scrutinized.

## Figures and Tables

**Table 1:** Patient characteristics.

		Total n/%	Patients		*p*-Value	Annual SPC	<1 SPC/ Year
			with TL	Without TL			
Participants [n]		115	53/46	62/54	0.240 *	77/67	38/33
Gender [n]	female	51/44	25/22	26/23	0.289 **	39/34	12/10
	male	64/56	28/24	36/31		38/33	26/23
Age (T0) [years]		54.9 ± 10.34					
Age (T1) [years]		55.4 ± 10.3	56.4 ± 9.5	54.5 ± 11.0	0.333 *	54.8 ± 10.1	56.6 ± 10.8
Age (T2) [years]		64.8 ± 9.85					
Initial periodontal diagnosis							
Stage III [n]		87/76	32/28	55/48	<0.001 ** (df = 2)	63/55	24/21
Stage IV [n]		28/24	21/18	7/6		14/12	14/12
Grading scale A		3/3	2/2	1/1	0.341 **	3/3	0/0
Grading scale B		66/57	28/24	38/33		44/38	22/19
Grading scale C		46/40	23/20	23/20		30/26	16/14
Step 1 + 2 duration [month]		4.2 ± 3.0	3.5 ± 2.3	4.9 ± 3.5	0.008 * (|r|= −0.46)	4.2 ± 2.8	4.3 ± 3.6
Risk factors at T1							
Active smoker		25/21	14/12	11/10	0.132 **	16/14	9/8
Former or non-smoker		90/78	39/34	51/44		61/53	29/25
Diabetes		12/10	6/5	6/5	0.388 **	10/9	2/2
Supportive periodontal care							
Patients with	annual SPC	77/67	35/30	42/37	0.338 **		
	<1 SPC/year	38/33	18/16	20/17			
SPC duration [years]		9.0 ± 2.4	9.0 ± 2.5	9.0 ± 2.3	0.495 *	8.9 ± 2.6	9.0 ± 1.8
Total number of SPC [n]		10.6 ± 3.8	10.4 ± 4.0	10.8 ± 3.6	0.549 *	11.0 ± 4.0	10.0 ± 3.3
Mean number SPC/year [n]		1.3 ± 0.5	1.2 ± 0.6	1.3 ± 0.5	0.676 *	1.3 ± 0.6	1.2 ± 0.4
Bone loss							
Max. periodontal BL [%] T0		45.4 ± 21.0	50.6 ± 21.0	41.0 ± 20.1	0.014 * (|r| = −0.54)	44.7 ± 20.9	46.8 ± 21.4

Abbreviations and notes: T0 = baseline, T1 = re-evaluation, T2 = last SPC visit, SPC = supportive periodontal care, TL = tooth loss, BL = bone loss, n = number of patients; * Pearson’s correlation coefficient; ** chi-square test.

**Table 2:** Tooth-specific characteristics at different time points.

	T0	T1	T2
Number of teeth	2647	2579	2446
per patient	23.202 ± 4.46	22.43 ± 4.92	21.27 ± 5.69
Tooth type [n/%]			
Anterior	1284/48.5	1266/49.1	1218/49.8
Premolar	746/28.2	731/28.3	691/28.2
Molar	617/23.3	582/22.6	537/22.0
FI [n/%]			
Single-rooted teeth	1852/70.1	1828/70.9	1753/71.6
Multi-rooted teeth	795/30.0	751/29.1	693/28.3
Without FI	482/18.2	593/22.9	439/17.9
With FI	313/11.8	158/6.1	254/10.4
Degree I	147/5.6	87/3.4	161/6.6
Degree II	106/4.0	40/1.5	74/3.0
Degree III	60/2.3	31/1.2	19/0.8
TM [n/%]			
Without mobility	2087/78.8	2224/86.2	2176/89.0
With mobility	560/21.2	355/13.8	270/11.0
Degree I	367/13.9	262/10.2	206/8.4
Degree II	150/5.7	82/3.2	56/2.3
Degree III	43/1.6	11/0.4	8/0.3
Abutment teeth [n/%]			
No abutment tooth	2255/85.2	2187/84.8	1971/80.6
Number of abutment teeth	392/14.8	392/15.2	475/19.4
Fixed	352/13.3	348/13.5	351/14.3
Removable	40/1.5	44/1.7	124/5.1
Max. number PD ≥ 6 mm [n/%]	838/31.67%	296/11.48%	302/12.35%
Mean PD [mm]	3.39 ± 1.19	2.78 ± 0.81	2.98 ± 0.80
Mean CAL [mm]	3.73 ± 1.87	3.03 ± 1.37	3.31 ± 1.39
Mean BOP [%]	31.74 ± 25.9	14.45 ± 17.18	16.83 ± 15.83

Abbreviations and notes: T0 = baseline, T1 = re-evaluation, T2 = last SPC visit, SPC = supportive periodontal care, FI = furcation involvement, TM = tooth mobility, PD = pocket probing depth, CAL= clinical attachment level, n = number of teeth, BOP = bleeding on probing.

**Table 3:** TL and PTL within 10 years after Step 2 (T1), taking into account FI, abutment tooth status, and TM at start of SPC.

	FI				Abutment Tooth Status				TM	
	Single-Rooted Teeth	Multi-Rooted Teeth			No Abutment Tooth	Total	Fixed	Removable	Teeth Without Mobility	Teeth with Mobility
		Total	Without FI	with FI						
Total										
n	1828	751	593	158	2187	392	348	44	2224	355
TL [n]	75	58	40	18	94	39	32	7	83	50
TL [%]	4.1	7.7	6.8	11.4	4.3	10.0	9.2	15.9	3.7	14.1
PTL										
TL [n]	70	48	34	14	84	34	28	6	68	50
TL [%]	3.8	6.4	5.7	8.7	3.8	8.7	8.0	13.6	3.1	14.1

Abbreviations and notes: TL = tooth loss, TM = tooth mobility, FI = furcation involvement, PTL = periodontal tooth loss.

**Table 4:** Multilevel logistic regression analysis [1st level: patient, 2nd level: tooth (T1)]: PTL during SPC, according to different risk factors at beginning of SPC (T1).

Parameters	Regression Coefficient	Standard Error	*p*-Value	OR	95% CI for OR	
					Lower Limit	Upper Limit
Constant	−8.466	1.471	<0.001	<0.001	1.176 × 10^−5^	0.004
Tooth level						
Abutment status						
Removable	0.590	0.665	0.375	1.803	0.489	6.645
Fixed	0.533	0.292	0.068	1.704	0.962	3.018
No abutment tooth	*reference*					
FI						
Degree III	1.957	0.837	0.019 *	7.076	1.371	36.529
Degree II	1.583	0.709	0.026 *	4.871	1.212	19.579
Degree I	0.801	0.551	0.146	2.229	0.756	6.569
Degree 0	*reference*					
TM						
Degree III	3.158	0.865	<0.001 *	23.534	4.309	128.547
Degree II	2.283	0.426	<0.001 *	9.810	4.259	22.597
Degree I	0.994	0.342	0.004 *	2.703	1.382	5.288
Degree 0	*reference*					
Mean PD	0.794	0.257	0.002 *	2.211	1.336	3.660
Mean CAL	−0.183	0.202	0.367	0.833	0.560	1.239
Patient level						
Age	0.038	0.021	0.065	1.039	0.988	1.083
SPC						
1 SPC/year	−0.511	0.394	0.194	0.600	0.278	1.297
<1 SPC/year	*reference*					
SPC appointments (n)	−0.014	0.050	0.784	0.986	0.894	1.088
Max. periodontal BL	0.017	0.009	0.054	1.018	1.000	1.036
Stage						
IV	1.078	0.423	0.011 *	2.940	1.282	6.740
III	*reference*					

*Dependent variable:* PTL during SPC (n = 2602 teeth), model was adjusted for SPC duration; −2 LL: 15,244.231. *Abbreviations and notes:* BL = bone loss, PTL = periodontal tooth loss, T1 = re-evaluation, FI = furcation involvement, TM = tooth mobility, SPC = supportive periodontal care, PD = pocket probing depth, CAL = clinical attachment level, OR = odds ratio, CI = confidence interval; * = statistically significant.

## Data Availability

The datasets generated and/or analyzed during this study are available from the corresponding author upon reasonable request.
